# Virtual Reality for Pediatric Sedation: A Randomized Controlled Trial Using Simulation

**DOI:** 10.7759/cureus.486

**Published:** 2016-02-09

**Authors:** Pavan P Zaveri, Aisha B Davis, Karen J O'Connell, Emily Willner, Dana A Aronson Schinasi, Mary Ottolini

**Affiliations:** 1 Division of Emergency Medicine, Children's National Health System; 2 Division of Hospital Medicine, Children's National Health System; 3 Division of Emergency Medicine, Lurie Children's Hospital

**Keywords:** virtual reality, pediatric procedural sedation, assessment, simulation

## Abstract

Introduction: Team training for procedural sedation for pediatric residents has traditionally consisted of didactic presentations and simulated scenarios using high-fidelity mannequins. We assessed the effectiveness of a virtual reality module in teaching preparation for and management of sedation for procedures.

Methods: After developing a virtual reality environment in Second Life® (Linden Lab, San Francisco, CA) where providers perform and recover patients from procedural sedation, we conducted a randomized controlled trial to assess the effectiveness of the virtual reality module versus a traditional web-based educational module. A 20 question pre- and post-test was administered to assess knowledge change. All subjects participated in a simulated pediatric procedural sedation scenario that was video recorded for review and assessed using a 32-point checklist. A brief survey elicited feedback on the virtual reality module and the simulation scenario.

Results: The median score on the assessment checklist was 75% for the intervention group and 70% for the control group (*P *= 0.32). For the knowledge tests, there was no statistically significant difference between the groups (*P *= 0.14). Users had excellent reviews of the virtual reality module and reported that the module added to their education.

Conclusions: Pediatric residents performed similarly in simulation and on a knowledge test after a virtual reality module compared with a traditional web-based module on procedural sedation. Although users enjoyed the virtual reality experience, these results question the value virtual reality adds in improving the performance of trainees. Further inquiry is needed into how virtual reality provides true value in simulation-based education.

## Introduction

Pediatric procedural sedation is a common intervention performed in multiple areas of the hospital. Competency assessment and sedation credentialing are largely determined by individual hospitals and their sedation committees, which are often chaired by an anesthesiologist whose reference standard may be limited to the operating room [1]. With procedural sedation occurring across hospital settings, training should reflect additional patient care areas.

In training health care providers in procedural sedation, as with all other clinical areas, multiple challenges exist, ranging from the new evidence to be incorporated into existing time-limited curricula to the need to improve not only knowledge but also technical skills and critical reasoning capabilities. Physician and nurses have historically been trained under an apprenticeship model in which they wait for an opportunity to perform a particular skill under the guidance of a senior faculty member [2]. This approach can result in far fewer practice occasions than desirable to achieve competence and may result in unnecessary discomfort for patients while trainees perform procedures they are not adept in.

In the past decade, simulation has emerged as a technique for practicing high-stakes, yet low-frequency tasks, in medical education curricula, including pediatric procedural sedation [3-5]. Many studies have demonstrated that virtual reality is well accepted, and some have demonstrated its validity for skills training, with particularly good evidence in surgical fields [6-11]. However, studies assessing its effectiveness in team training and patient care scenarios has been limited [12-14]. A recent meta-analysis by Cook indicated that virtual reality performed better than no intervention, but not better than other instructional designs [15]. The aim of this study was to develop a virtual reality–based procedural sedation experience and assess its effectiveness compared to an existing web-based didactic training module. Educational outcome measures included high-fidelity simulation and knowledge assessments.

## Materials and methods

### Study design

We conducted a randomized controlled trial to assess the effectiveness of the virtual reality educational module compared with the traditional web-based module for pediatric sedation training. We chose a high-fidelity simulation for assessment to have participants show how they perform procedural sedation. The intervention group used the virtual reality module, and the control group completed the web-based module. The study was granted exempt status by the Institutional Review Board at Children’s National Medical Center (#Pro00001633) with a waiver of written informed consent. The authors provided an information sheet to each participant prior to participation. We invited all postgraduate year two and three pediatrics residents to participate in the study during the 2012-2013 academic year, arranging to complete enrollment during their Emergency Department rotation. Postgraduate year one residents were excluded to minimize variation in experience. An information sheet was provided to all residents explaining the study. Once enrolled and scheduled, the resident was randomized to the intervention or control group by an administrative assistant. The participant completed the pretest in advance, spent 20 to 30 minutes to complete the intervention or control module, and then proceeded to the Simulation Center to complete the simulation assessment and post-test.

### Educational modules

**Figure 1:**
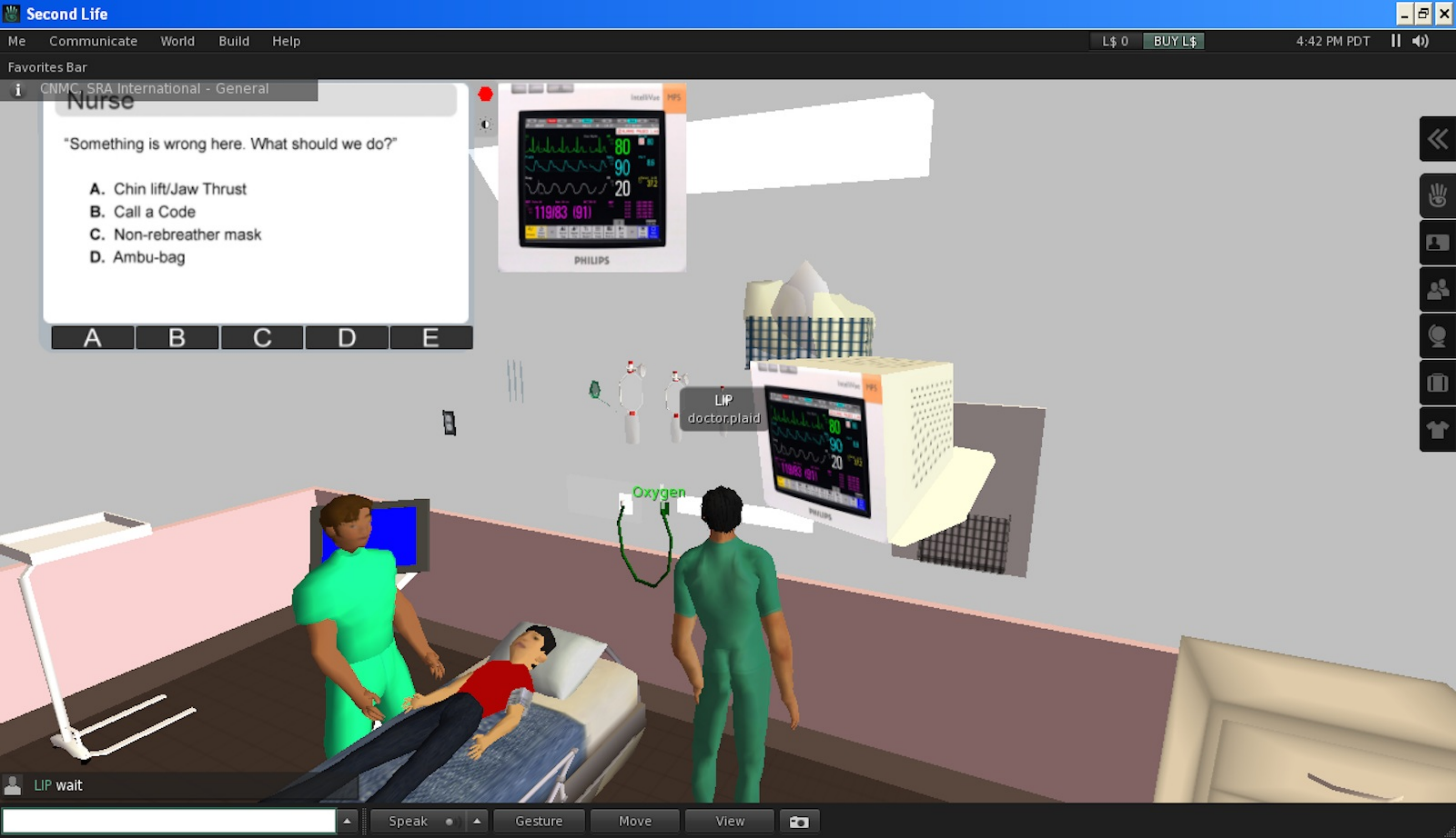
Virtual reality sedation environment

In the first phase of this project, in partnership with a technology consultant (SRA International), we developed a virtual reality program in which participants engage in the complete process of providing pediatric procedural sedation in two unique patient scenarios. At the time of development, the team chose Second Life® (Linden Lab, San Francisco, CA) as the software environment for this project. The virtual reality module provided an anteroom to orient the user to basic functions of movement and interaction in the Second Life environment. Then, the user proceeded to one room where a child required sedation for a fracture reduction and another room where a child required sedation for a CT scan with contrast. Each room was constructed similarly to the actual rooms at our hospital (Figure 1).

The user then responded to a series of questions to prepare the patient for sedation, worked with a pre-programmed nurse to administer the sedation, and needed to intervene and treat a complication related to the sedation. With a resolution of the complication, the scenario ended and the user was directed to the other room.

**Figure 2:**
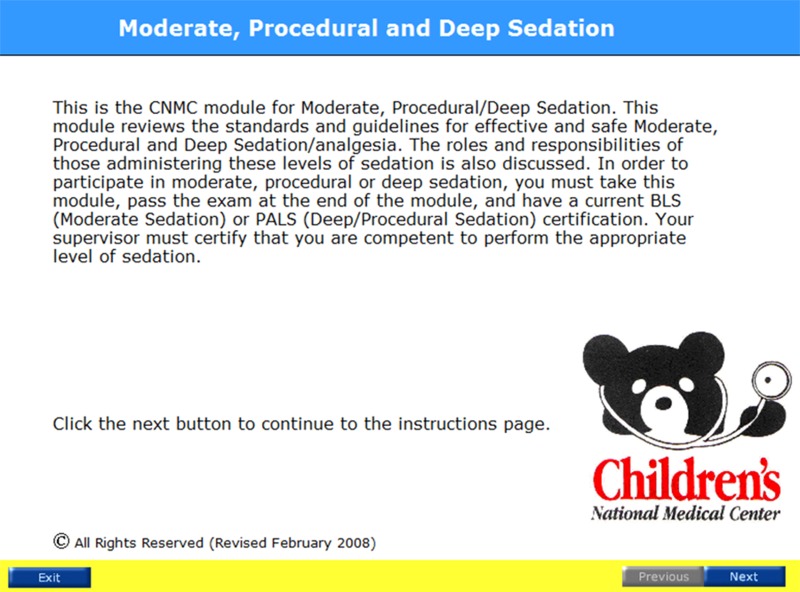
Control Module Used with permission from Dr. Richard Kaplan.

The control module was a web-based learning module with a series of slides with overlaid audio, intermittent video clips, and knowledge-check questions (Figure 2). The slides covered definitions, indications, and contraindications, a review of the medications, competencies of the providers, complications, equipment needed, and the process for sedation. This module was developed locally by the Department of Anesthesiology and has been the standard at our institution for credentialing in procedural sedation for many years.

### Simulation scenario

After completing the intervention or control module, all residents then immediately participated in a simulated pediatric procedural sedation scenario. All simulations occurred in the Simulation Center with an infant patient simulator (SimBaby, Laerdal Medical, Stavanger, Norway). A standard scenario and script were created and piloted before enrolling participants with a pediatric emergency medicine attending and a fellow. The scenario was standardized with two team members (AD, PZ) interacting with the participants following an explicit script. The Simulation Center was used to ensure a standard setup and availability of equipment, as well as familiarity with the mannequin and arrangement by the residents. The scenario involved a 12-month-old infant with bilateral lower-extremity burns requiring debridement. The scenario required answering several questions to set the stage followed by a preparation phase and complication phase. Once the requested medication was delivered, the patient would develop laryngospasm (if given ketamine) or hypopnea (if given a narcotic/benzodiazepine), both of which were programmed to resolve once positive-pressure ventilation was delivered.

### Assessment instruments

**Figure 3:**
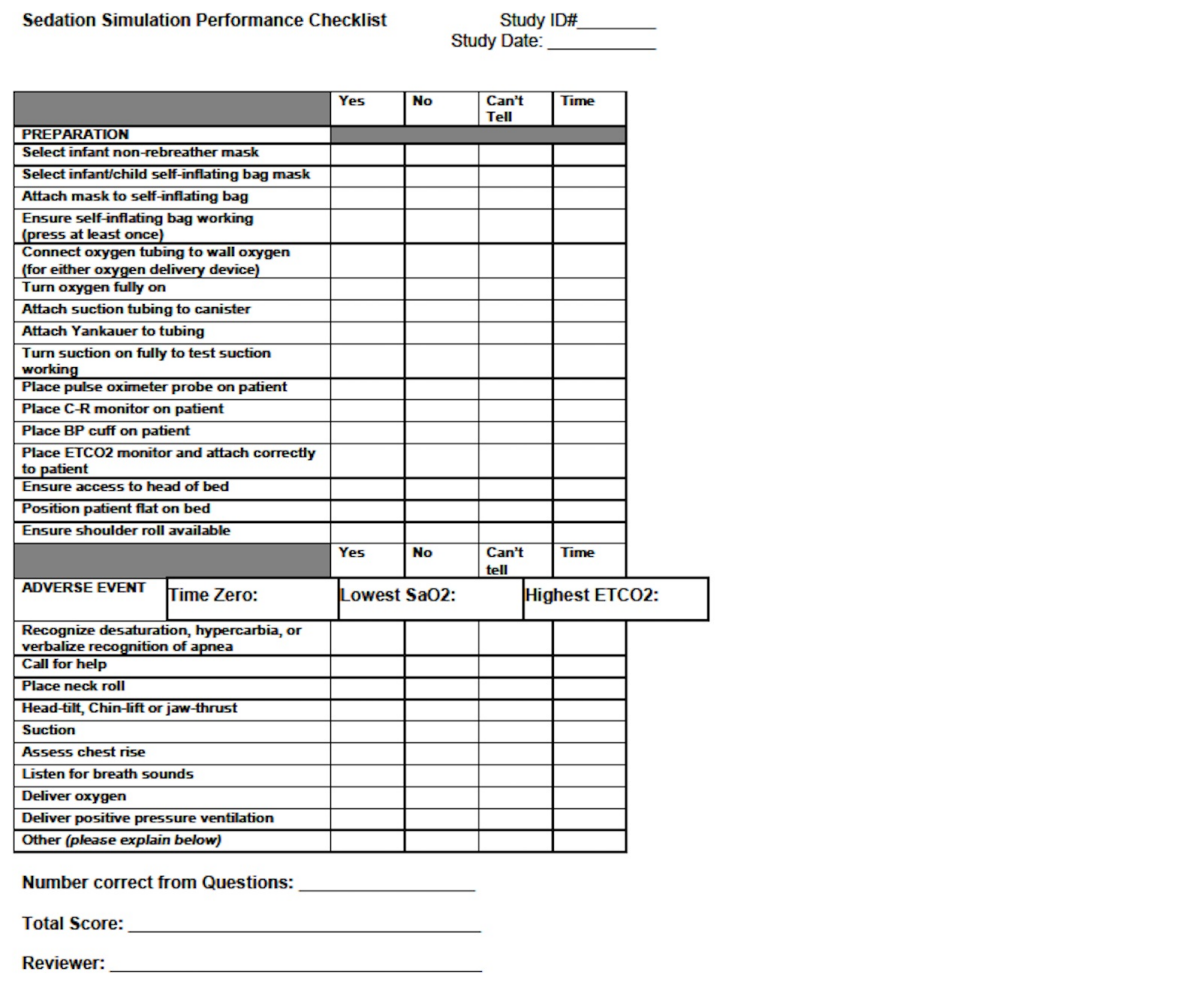
Sedation Simulation Checklist

The preparation and event were video and audio recorded in the Simulation Center. The video review was completed with the Laerdal Debrief Viewer (Laerdal Medical, Stavanger, Norway), which allowed three camera views, a monitor review, and an event log to be viewed simultaneously. Each performance video was reviewed by one or two team members (KO, EW) blinded to the group allocation. Performance on preparation and management of a complication was assessed using a 32-point checklist, adapted for this sedation scenario from a previously published checklist [16]. The initial checklist was determined by a consensus from a panel of experts in pediatric emergency medicine and pediatric procedural sedation [16]. The clinical performance checklist was first modified for the delivery of care as it occurs at our hospital. Then, in a modified Delphi format, the revised checklist was sent to a panel of experts in simulation, emergency medicine, and pediatric emergency medicine to ensure content validity in the simulated setting as well as clarity and applicability. With three rounds, a consensus was achieved on the checklist (Figure 3).

A twenty question test was administered to assess knowledge change. This test is the required assessment for the control module, which is currently required as part of credentialing at our hospital. The same test was used as the pre-test and the post-test with only a change in the order of the questions.

In addition to knowledge and performance assessment, feedback on the virtual reality module and the simulation scenario was obtained from each participant through a brief five-item survey.

### Statistical analysis

To demonstrate a 20% difference between scores on the performance checklist of the intervention and control groups, each group required 15 participants. A Mann-Whitney U test was performed to compare the intervention and control group scores on the checklist, the pre-tests, and the post-tests. A Wilcoxon signed rank test compared pre- versus post-test improvements for each group. Descriptive results of the feedback surveys on the virtual reality module and simulation were also reviewed. An intraclass coefficient was determined to assess the reliability of the video review process between the two reviewers.

Study data were collected and managed using REDCap electronic data capture tools hosted at Children's National Medical Center. REDCap is a secure, web-based application designed to support data capture for research studies [17]. Statistical analysis was performed using SPSS version 21 (SPSS, Inc., Chicago, IL).

## Results

**Figure 4:**
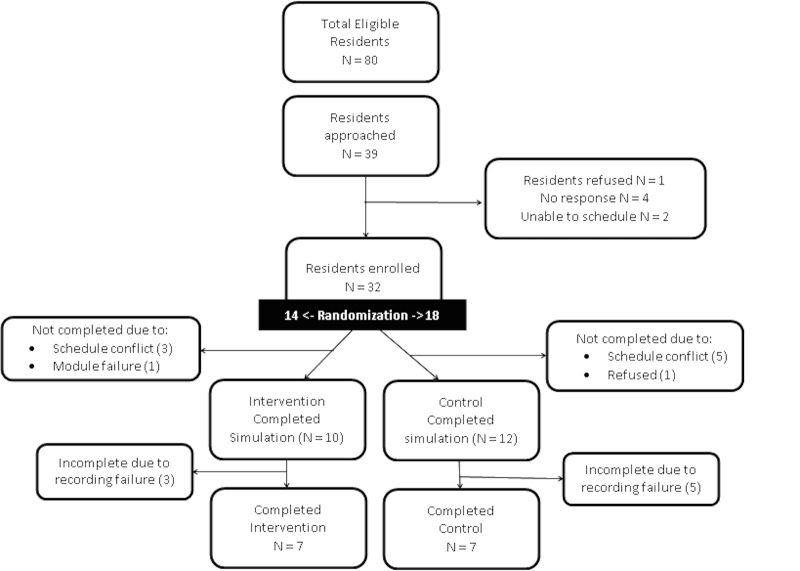
Study Enrollment

We enrolled 32 residents in this study from May to December 2013. Due to various conflicts, only 10 residents in the intervention group and 12 residents in the control group completed the simulation (Figure 4). Due to eight recording failures, simulation data for assessment of the primary outcome were obtained for only seven residents in each group. The groups were fairly similar in their level of training and experience with no statistically significant differences between the groups (Table 1).

**Table 1:** Participant Characteristics IQR = interquartile range. P value > 0.05 for all comparisons between groups using Mann-Whitney U test.

Characteristic	Intervention (N = 7)	Control (N = 7)*
PGY 2	4	5
Sedations participated in (median, IQR)	1 (1-5)	2 (2-2)
Total sedations performed as primary provider	4	7
Total sedation complications managed	2	2

### Assessment

Results of the pre-test, post-test, and simulation assessment are provided in Table 2. The intervention group had a median score of 75% for the assessment checklist versus 70% for the control with no statistically significant difference (*P *= 0.32). Reliability of the video review was confirmed with an intraclass coefficient of 0.69.

**Table 2:** Scores of Intervention and Control Groups *Based on Mann-Whitney U test for comparison of intervention and control groups +Based on Wilcoxon signed rank test for comparison of pretest to post-test score.

	Median (Interquartile Range)		*P * Value*
	Intervention Group	Control Group	
Knowledge pre-test	14/20 (13.5 to 15)	13/20 (11.75 to 14.25)	0.25
Assessment checklist	24/32 (22.5 to 25)	22.5/32 (20 to 25)	0.32
Knowledge post-test	15/20	16/20	
Difference, post-test – pre-test	1 (–1 to 2.25) *P = *0.13^+^	3 (1 to 4) *P *= 0.007^+^	0.14

For the knowledge test, there were no statistically significant differences between the groups for both pre- and post-test scores. The intervention group improved its score by a median of 1 point with an interquartile range of –1 to 1.25, whereas the control group improved its score by a median of 3 points with an interquartile range of 1 to 4 points.

### Usability of virtual reality module

In the brief survey of usability, eight of 12 participants who completed the virtual reality module agreed or strongly agreed that the module was easy to use and navigate. All residents who did the virtual reality module felt it added to their education regarding sedation. Residents further commented:

“I enjoyed the interactive display, allowed me to critically think and get instant feedback.”

“Overall, enjoyed the experience.”

“Lots of fun! Made learning easy, active, and interactive.”

“Great experience. Would love more of these throughout training.”

“Great module, would recommend it to all trainees.”

“Allowed for step-by-step thinking process in regards to approaching sedating a patient.”

## Discussion

This study demonstrated that residents performed similarly after review of a standard web-based module or a virtual reality module. Development of the module was a time- and fund-intensive investment occurring over two years of interactions with a software consultant and a $40,000 grant to create two scenarios. The assessment did not demonstrate any difference between the traditional web-based and virtual reality modules aside from a positive participant experience, although limited by an inadequate sample.

In looking at the value of virtual reality, insight is provided by analyzing studies by the level reached on the Kirkpatrick hierarchy. Kirkpatrick described level 1 of evaluation as reactions; level 2, learning; level 3, behavior and performance; and level 4, results—which would be patient outcomes in the clinical setting. Most studies have reported excellent results when assessing reaction at Kirkpatrick’s level 1 [6-7, 18-20], but few studies have shown behavioral outcomes at Kirkpatrick level 3 for virtual reality. Our study adds to the literature in showing positive findings at Kirkpatrick level 1 in that the participants enjoyed the experience; however, performance at levels 2 and 3 was no different than our control module, and, thus, it is unclear if we can improve performance beyond the control module through virtual reality.

Studies evaluating virtual reality to teach common procedures, such as intravenous catheter placement, fiberoptic intubation, and bronchoscopy, have reported improvement in performance success with decreased errors and time to completion [7-8, 11, 21]. In a review of the neurosurgical literature using virtual reality, most studies demonstrated positive user feedback with only a few able to show improvements in time to completion and one with performance improvement on a checklist [6]. When evaluating performance on specific surgical procedures after training with virtual reality, several studies demonstrated modest improvements on procedural checklists without any difference in time to completion [9-10, 22]. Kundhal, et al. correlated a task-based checklist from a virtual reality performance with actual operating room performance, an example starting to reach Kirkpatrick level 4 assessment [23]. These studies have shown that virtual reality training can effect change at Kirkpatrick level 3 and occasionally at level 4, patient outcomes, achieving demonstrable value to education.

However, those studies evaluating an interactive procedure, such as mass casualty triage, have had more mixed results, not offering enough argument to suggest widespread use and investment [12, 24-28]. Although most participants enjoy the experience, the performance measures do not uniformly improve over alternative modalities, such as live simulation [12, 24] or card sort triage [27]. Such results continue to call into question the true value of virtual reality.

Studies specifically evaluating Second Life as the platform for virtual reality have demonstrated feasibility, with positive participant feedback related to the modality, but have not researched performance outcomes [14, 18-20]. When Kidd, et al. used Second Life to teach nursing students how to do mental health assessments, the students enjoyed the safety of simulation and the interactivity afforded but dressing and maneuvering the avatars and a lack of realism limited full engagement [19]. At another nursing school, the faculty implemented Second Life for one curriculum, where again reactions were positive but limited by lack of orientation and difficulty with the technology [20]. Finally, in a third report, as part of a $1.6 million grant, the faculty developed a simulation instruction experience in Second Life and reported that it was feasible based on user feedback, but did not demonstrate any outcomes on assessment [14]. In contrast, Cohen, et al.’s development in Second Life demonstrated positive participant feedback and face validity, yet called for “robust assessment metrics,” highlighting the challenges in showing outcomes improvement [18].

Similar to our study where a scenario was to be managed, Nyssen, et al. demonstrated that the virtual reality environment can result in improved diagnosis time and treatment scores when repeating a virtual reality scenario, but can fall short in covering the full range of patient scenario management compared with high-fidelity simulation scenarios. The specific area of improvement was in technical skills, which prompts the question of whether the virtual reality was necessary or a reading or didactic may have sufficed [29]. In our study, looking at preparation for sedation and management of an adverse event, the virtual reality module did not demonstrate superior outcomes compared with the traditional web-based module.

The mixed results of studies evaluating the efficacy of virtual reality training on participant performance bring to the forefront the question of the value of virtual reality in medical education. Educators need to carefully assess the balance of cost, time, availability of outcome data, and generalizability to various settings and audiences before deciding to build or invest in virtual reality. Virtual reality as a modality to flip the classroom and immerse the learner has yet to be proven as creating value for the participant and the educator.

There were several limitations to this study. First, our study had a small number of participants in each group due to inadequate enrollment numbers and technical failures. Our planned recruitment numbers did not account for this unforeseen complication. This raises the possibility of our study failing to identify the effect of virtual reality due to an inadequate sample size. Second, we encountered funding shortages and were able to design only half of the intended Second Life scenarios. As others have shared in their descriptions of virtual reality development, the design and creation aspect can be quite expensive [14, 18, 20]. This can create significant challenges in creating a sustainable product and modifying it as users provide feedback. Fortunately, newer software for virtual reality function in common web browsers allows wider adaptability and easier modification. 

## Conclusions

Our study demonstrated high participant satisfaction with the virtual reality module, but we were unable to show a difference in sedation performance for those trained using virtual reality compared with the traditional web-based module. Participants felt the virtual reality environment added positively to their education and was easy to use. We acknowledge that our sample size for this research project was small and encourage further research to investigate the value of virtual reality modules in medical education. 
